# A practical framework for label-free GAG disaccharide analysis by HILIC-MS/MS: detailed protocols for analysis of diverse biological sample types

**DOI:** 10.1093/glycob/cwag104

**Published:** 2026-11-06

**Authors:** H Davies-Strickleton, George Taylor, James Allsey, Samie Dalgarno, Megan J Priestley, Isobelle Blair, Nabina Pun, Emily Williams, Magnus Nørregaard Nissen Grønset, Ivy Usanksy, Catherine B Lawrence, Rebecca L Miller, David Knight, Douglas P Dyer

**Affiliations:** Manchester Cell-Matrix Centre, Faculty of Biology, Medicine and Health, University of Manchester, M13 9PT, United Kingdom; Biological Mass Spectrometry Core Facility, Faculty of Biology, Medicine and Health, Manchester Academic Health Science Centre, University of Manchester, Manchester, M13 9PT, United Kingdom; Biological Mass Spectrometry Core Facility, Faculty of Biology, Medicine and Health, Manchester Academic Health Science Centre, University of Manchester, Manchester, M13 9PT, United Kingdom; Biological Mass Spectrometry Core Facility, Faculty of Biology, Medicine and Health, Manchester Academic Health Science Centre, University of Manchester, Manchester, M13 9PT, United Kingdom; Manchester Cell-Matrix Centre, Faculty of Biology, Medicine and Health, University of Manchester, M13 9PT, United Kingdom; Lydia Becker Institute of Immunology and Inflammation, MRC-UK Centre of Research Excellence in Exposome Immunology, Faculty of Biology, Medicine and Health, University of Manchester, M13 9PT, United Kingdom; Koch Institute for Integrative Cancer Research, Massachusetts Institute of Technology, Cambridge, MA, MA 02139, United States; Manchester Cell-Matrix Centre, Faculty of Biology, Medicine and Health, University of Manchester, M13 9PT, United Kingdom; Lydia Becker Institute of Immunology and Inflammation, MRC-UK Centre of Research Excellence in Exposome Immunology, Faculty of Biology, Medicine and Health, University of Manchester, M13 9PT, United Kingdom; Centre for Immuno-Oncology, Nuffield Department of Medicine, University of Oxford, Oxford, OX3 7DQ, United Kingdom; Manchester Cell-Matrix Centre, Faculty of Biology, Medicine and Health, University of Manchester, M13 9PT, United Kingdom; Copenhagen Center for Glycocalyx Research, Department of Cellular and Molecular Medicine, Faculty of Health Sciences, University of Copenhagen, Blegdamsvej 3, Copenhagen N DK-2200, Denmark; Division of Neuroscience, Geoffrey Jefferson Brain Research Centre Faculty of Biology, Medicine and Health, Manchester Academic Health Science Centre, The University of Manchester, Manchester, M13 9T, United Kingdom; Division of Neuroscience, Geoffrey Jefferson Brain Research Centre Faculty of Biology, Medicine and Health, Manchester Academic Health Science Centre, The University of Manchester, Manchester, M13 9T, United Kingdom; Copenhagen Center for Glycocalyx Research, Department of Cellular and Molecular Medicine, Faculty of Health Sciences, University of Copenhagen, Blegdamsvej 3, Copenhagen N DK-2200, Denmark; Biological Mass Spectrometry Core Facility, Faculty of Biology, Medicine and Health, Manchester Academic Health Science Centre, University of Manchester, Manchester, M13 9PT, United Kingdom; Manchester Cell-Matrix Centre, Faculty of Biology, Medicine and Health, University of Manchester, M13 9PT, United Kingdom; Lydia Becker Institute of Immunology and Inflammation, MRC-UK Centre of Research Excellence in Exposome Immunology, Faculty of Biology, Medicine and Health, University of Manchester, M13 9PT, United Kingdom; Division of Neuroscience, Geoffrey Jefferson Brain Research Centre Faculty of Biology, Medicine and Health, Manchester Academic Health Science Centre, The University of Manchester, Manchester, M13 9T, United Kingdom

**Keywords:** disaccharide analysis, glycosaminoglycan, HILIC-MS/MS

## Abstract

The extracellular matrix (ECM) and cell surface glycocalyx are key components of biology and play crucial roles in development and tissue function, as well as disease. Proteoglycans, and their glycosaminoglycan (GAG) side chains, are critical components of the ECM and the glycocalyx. GAGs can bind to many different proteins, such as chemokines, and form hydrated barriers around cells. Existing and new methods are helping us to uncover more about the roles of GAGs in biology. Here, we build upon these technologies by providing a streamlined, standardised, and extensively documented end-to-end workflow for GAG disaccharide analysis that can be readily implemented in standard analytical facilities for the analysis of diverse biological sample types. Comprehensive step-by-step guidance is provided for sample disruption, GAG disaccharide preparation from biological samples, and HILIC-MS/MS analysis. In addition, we demonstrate the broad applicability of the workflow across a diverse range of low-input biological samples, including cell pellets, FACS-sorted cells, frozen tissue, FFPE sections, serum, and mucus washes. By complementing existing and emerging analytical approaches, this workflow supports wider adoption of GAG disaccharide analysis and facilitates investigation of GAG function across diverse biological systems. This method will sit alongside existing and new techniques to help improve access to GAG analysis, and thereby further the field of understanding GAG function in complex biological contexts.

## Introduction

The extracellular matrix (ECM) and cell surface glycocalyx (the pericellular matrix that surrounds cells) are important structures across biological contexts from development of tissues to removal of invasive pathogens (Afratis et al. 2017; Gray et al. 2022; Varki et al. 2022; Sutherland et al. 2023). Proteoglycans, which are present on all mammalian cells to different extents, are one of the key components of the ECM and glycocalyx. Proteoglycans have crucial roles in tissue development and function (Schwartz and Domowicz 2018), but also in inflammatory based diseases (Gray et al. 2022) and cancer (Ahrens et al. 2020). Classically their function has been primarily focused on their role in forming the basement membrane, underlying endothelial and epithelial cell structures, and as part of the interstitial matrix, that is key to tissue formation. More recent studies have also localised key proteoglycan function to their presence on the cell surface, e.g. endothelial (Larsen et al. 2025) and immune cells (Priestley et al. 2025), as part of the cell surface glycocalyx (Sutherland et al. 2023).

Proteoglycans are formed by protein cores that can be secreted or embedded into the cell membrane (e.g. syndecans) that have glycosaminoglycan (GAG) side chains attached (Couchman and Pataki 2012). There are a number of different GAGs based on their sequence including, heparan sulfate (HS), chondroitin sulfate (CS) and dermatan sulfate (DS), in addition to the non-sulfated GAG hyaluronan (HA) (Fig. 1A) (Varki et al. 2022). HS is composed of repeating units of glucosamine (GlcN) and glucuronic/iduronic acid (GlcA/IdoA). CS chains are composed of repeating units of N-acetyl galactosamine (GalNAc) and GlcA, whilst DS incorporates GalNAc, IdoA and GlcA. HS, CS and DS structures are also differentially sulfated during their synthesis to produce charge, and potentially high degrees of chain specificity (Fig. 1A). HA, on the other hand, is not protein anchored and is composed of repeating units of N-acetyl glucosamine (GlcNAc) and GlcA.

**Figure 1:**
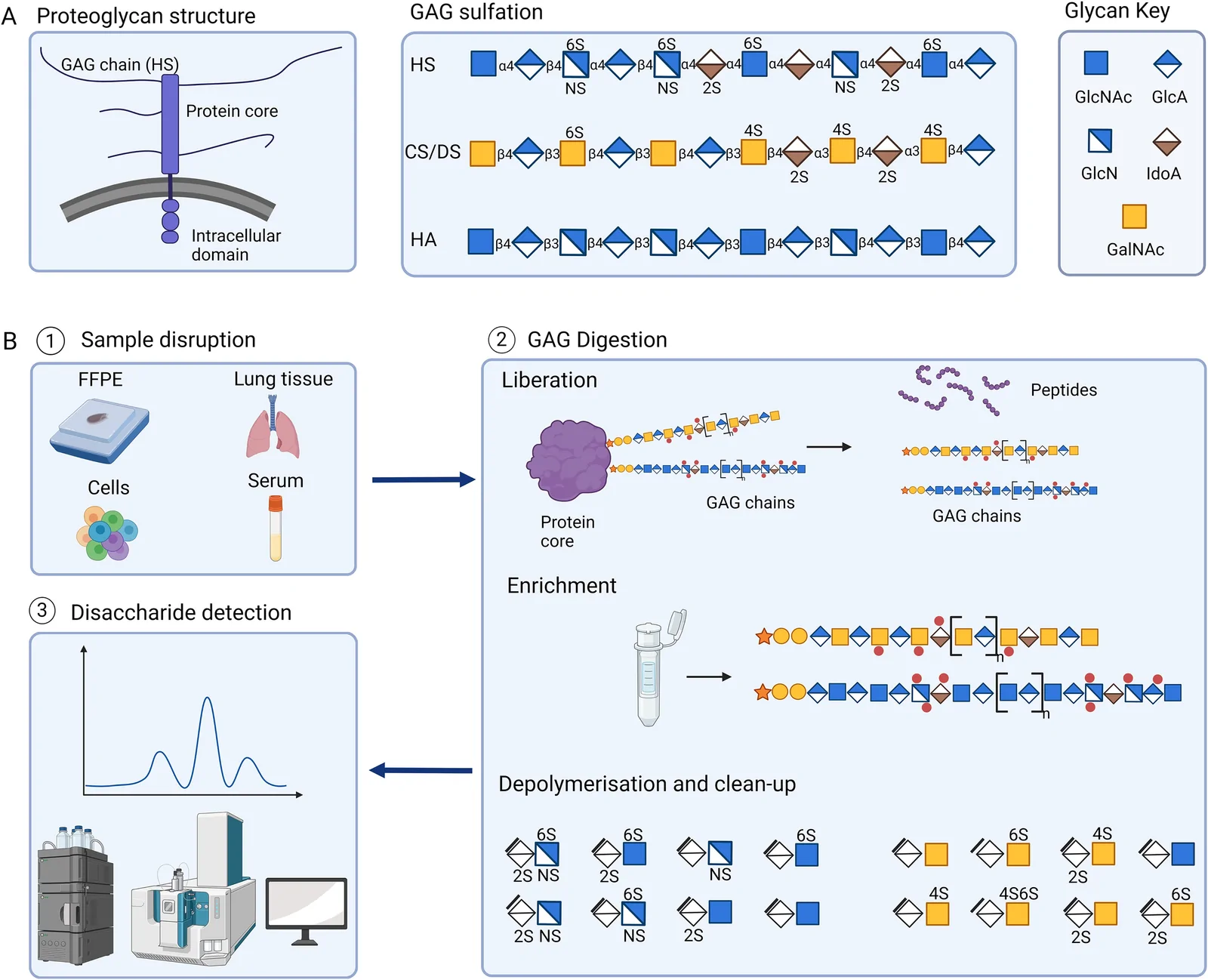
Overview of label-free GAG disaccharide analysis. (A) HS is composed of repeating units of glucosamine (GlcN) and glucuronic/iduronic acid (GlcA/IdoA). CS chains are composed of repeating units of N-acetyl galactosamine (GalNAc) and GlcA, whilst DS incorporates GalNAc, IdoA and GlcA. HA is composed of repeating units of N-acetyl glucosamine (GlcNAc) and GlcA. HS, CS and DS are highly sulfated, whilst HA is non-sulfated. (B) Schematic overview of label-free GAG disaccharide analysis. Sample disruption of diverse biological samples is followed by GAG liberation, enrichment and depolymerisation to yield disaccharides. Label-free disaccharides are analysed by HILIC-MS/MS. Figure created with BioRender.com.

The sulfated GAGs are critical to proteoglycan function due to their large size, hydrophillic nature and negative charge. These properties of the GAG produce hydrated adhesion-resistant structures that form barriers on the cell surface (Shi et al. 2025) and regulate their interaction with an array of proteins, including growth factors and chemotactic cytokines (Gray et al. 2023; Ridley et al. 2023). Despite their crucial biological function, the study of GAGs in complex biology has been limited by a lack of accessible tools for analysis in biologically relevant contexts.

Recently a number of new approaches have been developed to counter this problem. This includes the establishment of bump-hole engineering to allow “click-chemistry” based labelling of proteoglycans in vitro (Li et al. 2026). Furthermore, ToF-SIMS mass spectrometry is also in development to provide spatial information for GAG analysis using specialised apparatus (Milne et al. 2026). Alongside these new developments, the classic approach to analyse GAGs from biological samples has been to purify them, treat with GAG lyases to digest their chains into disaccharides and derivation (e.g. AMAC, 2-AB, Bodipy, Aniline) prior to analysis via several analytical techniques, such as HPLC or LC–MS (Skidmore et al. 2006; Lawrence et al. 2008; Lu et al. 2010; Wei et al. 2011; Yang et al. 2012; Gill et al. 2013; Volpi et al. 2014; Silva et al. 2020; Wang et al. 2020b; Basu and Weiss 2023; Karlsson et al. 2024; Basu et al. 2025).

Derivatisation-free disaccharide analyses have also emerged, offering benefits such as streamlined sample preparation and omitting the need for additional reagents that can present practical and safety considerations for some laboratories, including those in UK academic institutions. For the analysis of label-free GAG disaccharides, hydrophilic interaction liquid chromatography (HILIC)-based analyses have become an important platform. A number of applications have been demonstrated for label-free GAG disaccharide analysis by HILIC-analytics, including analysis of: purified GAGs by HILIC-MS (Gill et al. 2013; Poyer et al. 2021; Hirai et al. 2025); tissue sections by nano-HPLC–MS and homebuilt HILIC-WAX columns (Turiák et al. 2018; Raghunathan et al. 2019; Mernie and Zaia 2026); and laser microdissected tissue via HILIC coupled with cyclic ion mobility mass spectrometry (Mernie and Zaia 2026). These articles highlight label-free HILIC-MS as an attractive method for the study of biological samples at small scale.

Here, we aim to build on the existing methods to develop extensively documented end-to-end biological workflows that can incorporate diverse, small-scale biological samples and be readily implemented in standard analytical facilities, to increase the accessibility of GAG disaccharide analysis to non-specialist users. To this end, we: (a) describe a streamlined and standardised sample preparation protocol for label-free disaccharides, utilising commercially available consumables and without derivatisation steps or reagents that carry practical/safety concerns, ensuring the method can be performed and reproduced across laboratories including UK academic institutions; (b) employ HILIC-MS/MS on non-specialised, commonly available analytical equipment that is likely to be available to many researchers or be found within standard analytical facilities, ensuring analytical accessibility; (c) demonstrate broad applications by examination of a wide range of small-scale diverse biological sample types, including: cell culture pellets, FACS-sorted cells, frozen tissue, FFPE tissue sections, serum and mucus washes; (d) present an extensive set of detailed step-by-step guides of every step from sample disruption to data interpretation via protocols.io (https://doi.org/10.17504/protocols.io.x54v956n1l3e/v1) for high reproducibility and lower barriers to entry.

## Results

### Label-free, non-toxic, GAG disaccharide generation

Given the recent breakthroughs in understanding the wider biological functions of GAGs, we endeavoured to further enhance researcher/clinician access to GAG disaccharide analysis of diverse biological samples. We develop a streamlined and standardised method, supported by extensive step-by-step guides (https://doi.org/10.17504/protocols.io.x54v956n1l3e/v1) and demonstrate applicability to a wide range of biologically relevant samples, such as tissue biopsies, FFPE tissue, FACS sorted cells and serum. The methodology employs sample disruption, followed by GAG liberation, enrichment and depolymerisation into label-free disaccharides, which are then analysed by HILIC-MS/MS (Fig. 1).

The initial sample disruption was tailored to the sample type, with detailed step-by-step protocol steps documented (https://doi.org/10.17504/protocols.io.5jyl88pwrl2w/v1). Disruption tools employed included bead homogenisation of FFPE tissue, dry pulverisation of frozen tissue via cryoPREP and sonication for cell lysis. Following disruption, sample preparation procedures were designed to utilise commercially available consumables for streamlined robustness and reproducibility, and each step was extensively documented within a sample preparation protocol (https://doi.org/10.17504/protocols.io.kqdg31yxel25/v1). Within the sample preparation protocol, lysed samples were digested with pronase for the enzymatic degradation of proteins into peptides, and DNase for the digestion of DNA into nucleotides. The resulting small peptides and nucleotides, could then be separated from the large GAGs by size separation with a molecular weight cut off (MWCO) filter and centrifugation. Enriched GAGs were depolymerised with heparinases and/or chondroitinase ABC digestion, as per traditional ways of generating HS and CS/DS/HA disaccharides, respectively (Karlsson et al. 2021). Here, GAG disaccharides remained label-free, to reduce further sample processing, labelling inefficiencies, and practical/safety considerations from additional reagents. Disaccharides were then cleaned-up by solid phase extraction prior to HILIC-MS/MS analysis.

### HILIC-MS/MS detection of label-free GAG disaccharides

For the detection of label-free GAG disaccharides, HILIC was employed based on its ability to separate highly polar compounds, and was coupled to tandem mass spectrometry to provide specificity and sensitivity. Our methods for HILIC-MS/MS were designed to be carried out on standard analytical equipment and steps are described in extensive detail within associated protocols that detail the vialling of samples and standards (https://doi.org/10.17504/protocols.io.3byl46progo5/v1) and data acquisition parameters and data processing (https://doi.org/10.17504/protocols.io.n92ld6keog5b/v1).

First, using pure disaccharide standards and an internal disaccharide standard (the synthetic disaccharide ∆UA-2S GlcNCOEt-6S (Iduron, UK), HILIC-MS/MS conditions were developed for the unique detection and quantification of each disaccharide using MS/MS fragment ion intensities, with separate acquisition methods for HS and CS/DS/HA. Representative precursor ion and MS/MS spectra are shown in Supplementary Figs. 1 and 2, and ion transitions summarised in Supplementary Tables 1 and 2).

Isomeric disaccharides were differentiated through chromatographic separation where possible, while diagnostic MS/MS fragment ions were identified for isomers that could not be resolved chromatographically (Fig. 2). For instance, HILIC chromatography resolved several isomers including HS disaccharides ∆UA,2S-GlcNS and ∆UA-GlcNS,6S, which eluted at ~16.5 and 18 minutes, respectively (Fig. 2A). Similarly, for CS/DS/HA, the HA disaccharide ∆UA-(ß1-3)-GlcNAc and the CS/DS disaccharide ∆UA(1-4)-GalNAc separated at ~16 and 19 minutes, respectively (Fig. 2B). In these cases, chromatographic separation provided sufficient selectivity to distinguish the isomers, which were subsequently quantified using MS/MS fragment ion intensities (Supplementary Tables 1 and 2). Where isomeric disaccharides could not be fully resolved chromatographically, they were distinguished using diagnostic MS/MS fragment ions. For instance for HS, ∆UA,2S-GlcNAc and ∆UA-GlcNAc,6S showed overlapping retention windows (Fig. 2A), and so were distinguished by unique MS/MS fragments (236.9712 m/z for ∆UA,2S-GlcNAc; and 175.0256, 357.0148, 300.0403 m/z for ∆UA-GlcNAc,6S, Supplementary Table 1 and 2).

**Figure 2:**
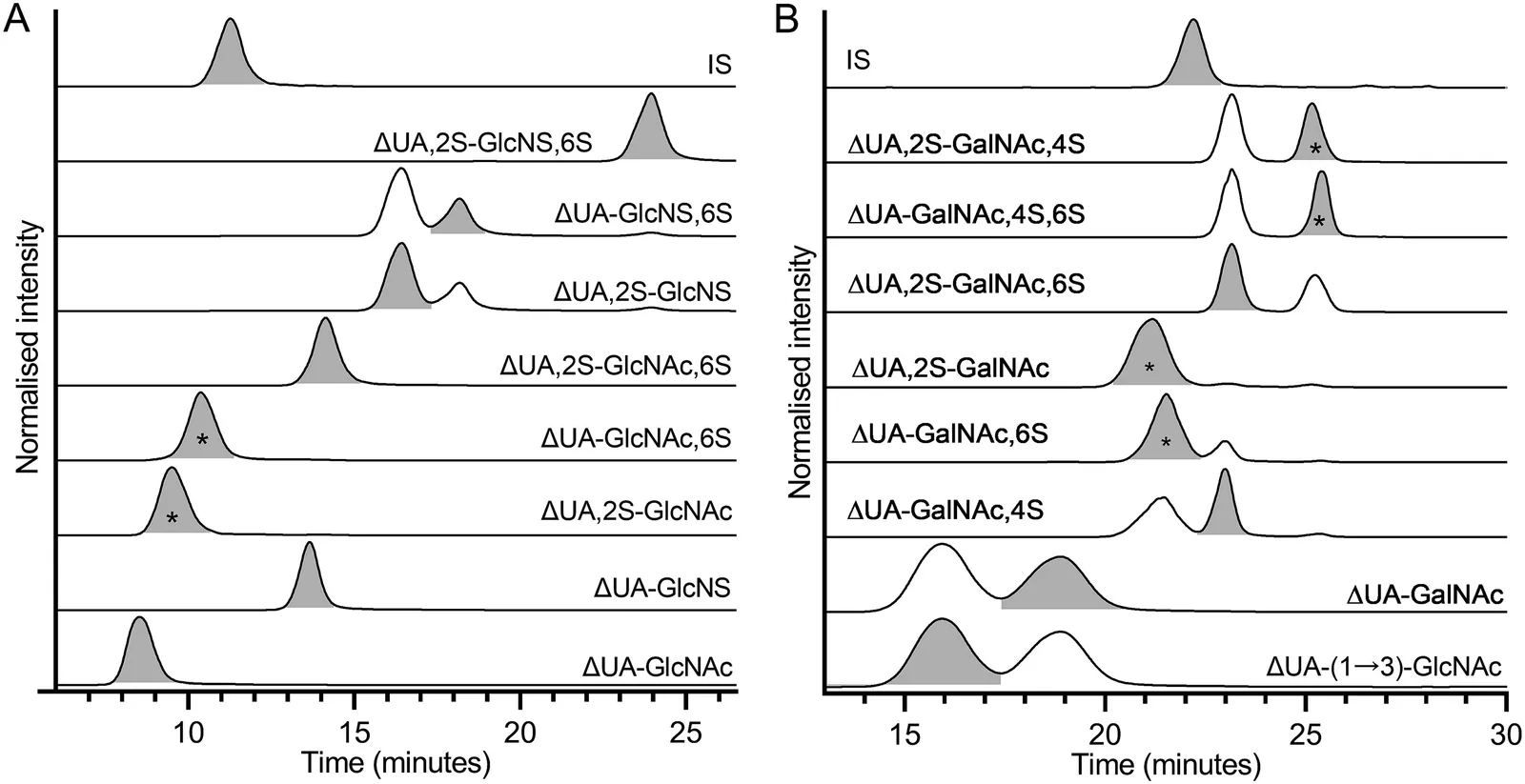
MS/MS extracted ion chromatograms of GAG disaccharide standards. Representative MS/MS extracted ion chromatograms obtained by HILIC-MS/MS analysis of equimolar standards of (A) HS disaccharides and (B) CS/DS/HA disaccharides. Shaded peaks indicate signals corresponding to the indicated analyte, whereas unshaded peaks represent chromatographically separated isomers. Asterisks indicate isomers that were not chromatographically resolved and were instead distinguished by diagnostic MS/MS fragment ions, as detailed in supplementary tables 1 and 2.

For all analytes, where more than one fragment ion was available, the corresponding signals were summed in order to improve sensitivity. Integrated peak area ratios (relative to the internal standard peak area) were calculated for each disaccharide. The internal standard (the synthetic disaccharide ∆UA-2S GlcNCOEt-6S, Iduron, UK, also used elsewhere (de Ruijter et al. 2012, Wang et al. 2020a)), served to account for variability in sample processing and instrument performance. All disaccharides produced measurable signals at the lowest amount of standard evaluated (0.025 pmol), with the exception of ΔUA,2S-GlcNAc,6S, which was measurable at 0.25 pmol due to background interference from the internal standard (Supplementary Figs. 3 and 4). In addition, all disaccharide standards demonstrated good response linearity across the evaluated range (0.025-5 pmol). While formal limits of detection were not determined, the lowest amounts evaluated were comparable to the sensitivity reported for other HILIC-MS-based GAG disaccharide methods (Hirai et al. 2025).

For the relative quantification of GAG disaccharides in biological samples, a semi-quantitative approach was implemented. Sample peak area ratios were normalised to that of an equimolar disaccharide standard (an equimolar mixture of HS or CS/DS/HA disaccharides—detailed steps for which are provided as a protocol (https://doi.org/10.17504/protocols.io.n92ld6knxg5b/v1). This normalisation corrected for disaccharide specific variability in analysis (e.g. ionisation efficiencies) and provided a single point calibration to approximate disaccharide amounts (semi-quantification). This approach is appropriate for the relative quantification of disaccharide amounts and total GAG amounts (by summation of disaccharide amounts), and the calculation of disaccharide composition (relative ratio of disaccharides compared to total GAG). Thus, providing an efficient tool for the exploration of GAG changes in a variety of biological contexts.

### Robust GAG disaccharide analysis across diverse biological sample types and input amounts

Given the need to determine the function of GAGs across different biological contexts (e.g. sample types and disease states) we used the label-free GAG disaccharide preparation and HILIC-MS/MS analysis to investigate GAG profiling of diverse biological sample types, including cell culture pellets, FACS-sorted cells, tissue specimens, FFPE scrolls and serum. Whilst analysing these diverse sample types, we utilised sample sizes to reflect those relevant to laboratory scale, and assessed method validity, including robustness/precision and accuracy.

Precision was first explored, in order to assess the ability of the method to analyse biological samples in a robust manner. Measurement of samples of mouse lung tissue homogenate prepared in multiple replicates was performed (Fig. 3, Supplementary Table 3; intra-sample precision). Disaccharide amounts and total GAG amounts demonstrated low Coefficient of Variation (CV) of 2–30%, indicating high consistency and intra-sample precision. For disaccharide composition (relative ratio of disaccharides compared to total GAG), intra-sample precision was also high, with CV of 1–18%. Therefore, the method has the necessary robustness for the routine analysis of biological samples. The GAG disaccharide compositions observed here were also in good agreement with those reported for bulk mouse lung tissue elsewhere (Supplementary Table 3, (Tykesson et al. 2025). Interestingly, comparison with this published dataset also highlighted the influence of sample preparation on total GAG recovery. While lung homogenates analysed here contained broadly similar amounts of HS, CS/DS and HA, bulk (whole) lung tissue analyses reported here and by Tykesson et al. showed relatively higher levels of HS and CS/DS compared with HA (Supplementary Fig. 5). This difference likely reflects the removal of insoluble material (with a higher HS and CS/DS content) during homogenate preparation, whereas bulk tissue analysis captures the entire tissue compartment. Together, these findings demonstrate both the precision of the method and the importance of considering sample preparation strategy when comparing GAG abundances between studies.

**Figure 3:**
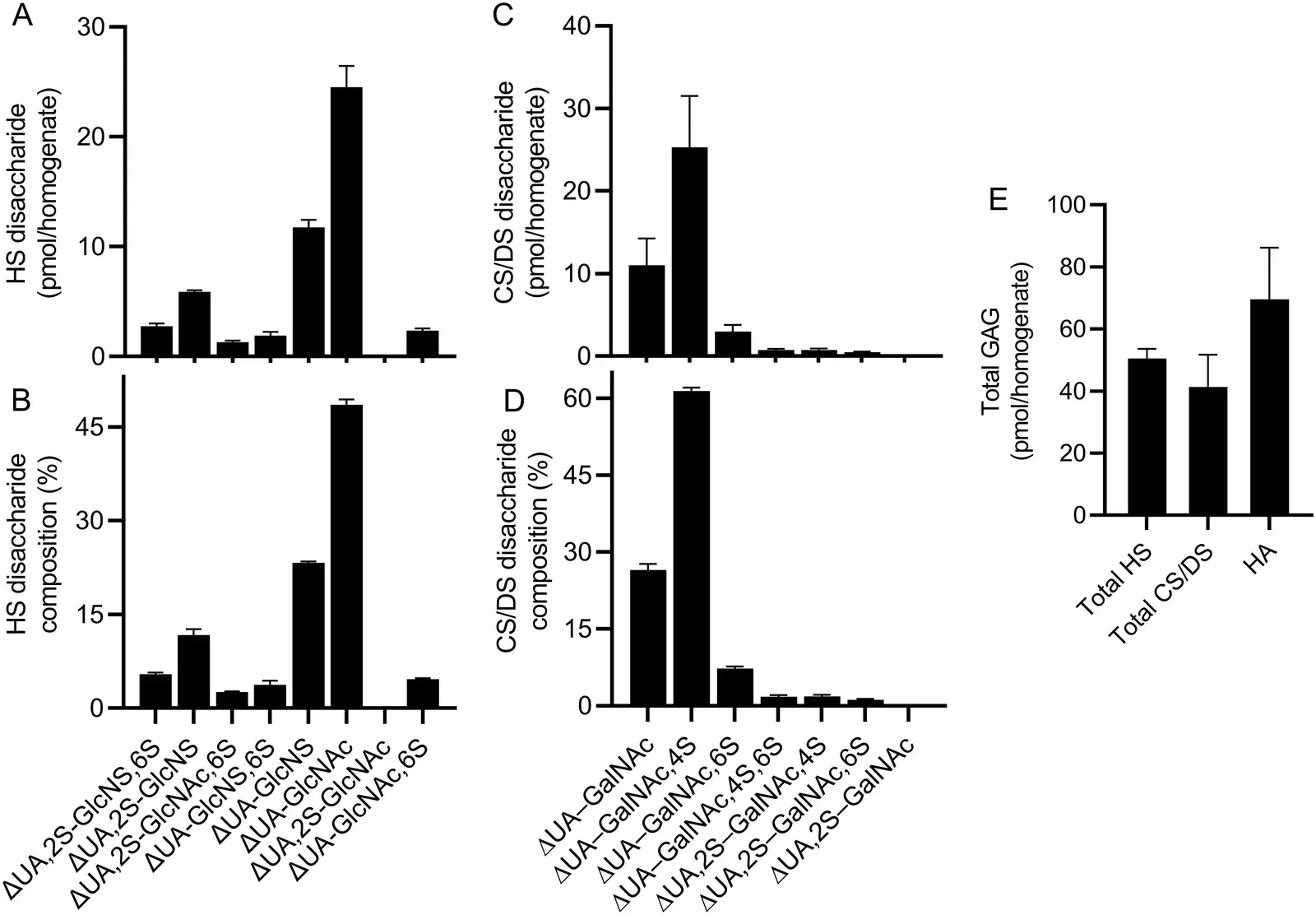
Robust detection of GAG disaccharides from tissue homogenates. Samples of mouse lung tissue homogenate were prepared in replicate and analysed by HILIC-MS/MS for HS disaccharides (A and B) and CS/DS/HA disaccharides (C and D). Disaccharide amount and total GAG amount were determined (A, C, and E), and disaccharide compositions calculated (B and D). (*n* = 5 replicates of the same sample, measured in a single experiment; intra-sample precision).

Next, CHO cell pellets, which have been well characterised previously (Chen et al. 2018; Miller et al. 2020; Karlsson et al. 2021), were analysed to assess the consistency of disaccharide detection across varying amounts of starting material. The disaccharide composition was in good agreement with previous reports using traditional HPLC-FLD methodologies for both HS and CS/DS disaccharides, further demonstrating the accuracy of the method for well documented biological samples (Chen et al. 2018; Karlsson et al. 2021) (Supplementary Table 4, data from 1e^6^ cells).

We then examined how this consistency was impacted by the number of cells per sample. As the number of cells per sample was reduced, the HS disaccharide composition remained largely consistent down to 2e^4^ cells (Fig. 4A). In contrast, the CS/DS disaccharide composition was dominated by a single disaccharide (93% ∆UA-GalNAc,4S, based on data from 1e^6^ cells) and the less abundant disaccharides were not detected in samples of cell number below 1e^6^ cells, yielding disaccharide compositions of 100% ∆UA-GalNAc,4S for samples of lower cell numbers (2e^4^ – 1e^5^ cells, Fig. 4B). Despite this, across samples of 2e^4^ – 1e^6^ cells, the disaccharide amounts and total GAG amounts scaled linearly with the number of cells per sample for both HS (r^2^ > 0.99) and CS/DS (r^2^ > 0.99) data (Fig. 4C–F). HA was not detected. Together, the CHO cell data demonstrate that the method can reproduce information from GAG sulfation for biological samples reported elsewhere, can provide relative comparison of total GAG amounts (and the most abundant individual disaccharides) from different amounts of starting material, and can utilise even low amounts of starting material.

**Figure 4:**
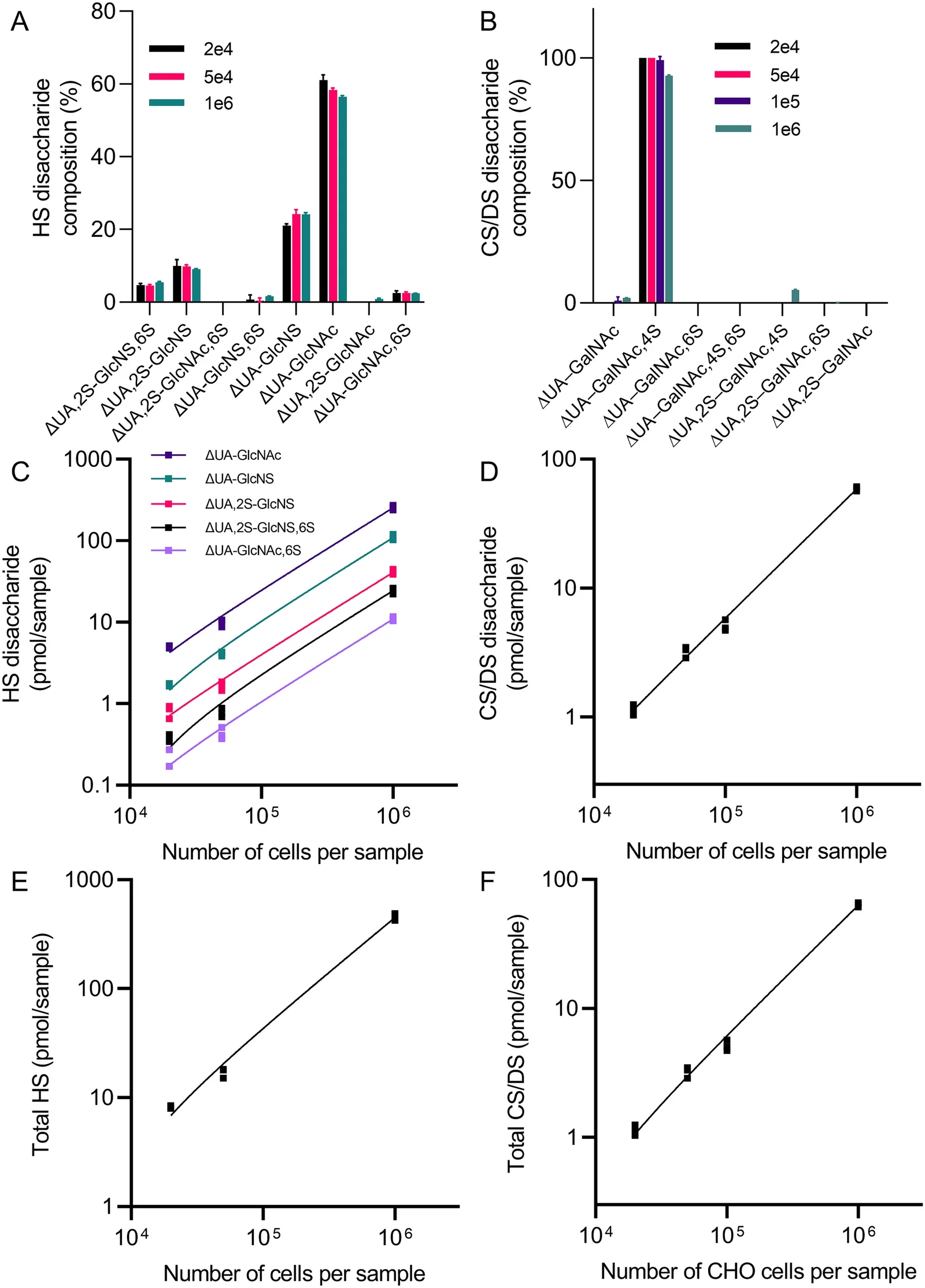
GAG analysis of WT CHO cells correlates with the number of cells analysed. Disaccharide composition for HS (A) and CS/DS (B) from different starting numbers of CHO WT cells: 2e^4^ (black), 5e^4^ (pink), 1e^5^ (blue, CS/DS only) and 1e^6^ (green) CHO cells (*n* = 3 replicates per sample group). (C) HS and (D) CS/DS/HA disaccharide amounts detected (pmol/sample) from different starting numbers of CHO cells. Data are shown for disaccharides detected in all sample groups; for CS/DS disaccharides this was only ∆UA-GalNAc. (E) HS and (F) CS total GAG detected (pmol/sample) from different starting numbers of CHO cells. All data (C-F) were fitted with a linear regression (weighting 1/x) and gave r^2^ > 0.99.

To further assess the applicability of the method to low-input cellular samples, cells were isolated from animal specimens and sorted into defined cell populations using fluorescence-activated cell sorting (FACS; Fig. 5, Supplementary Tables 5 and 6). The average number of cells isolated and used for GAG disaccharide analysis was 1260 (CS/DS/HA analysis of blood-derived monocytes), 11,344 (HS analysis of blood-derived monocytes), 2620 (CS/DS/HA analysis of brain-derived endothelial cells) and 23,580 (HS analysis of brain derived endothelial cells). GAG disaccharide analysis demonstrated that the most abundant disaccharides (HS disaccharides ∆UA-GlcNAc and ∆UA-GlcNS; and CS/DS disaccharides ∆UA-GalNac,4S and ∆UA-GalNAc,6S) were consistently detected across the 5 biological replicates measured (Supplementary Tables 5 and 6). It was noted that the level of HS and CS/DS disaccharides per cell was higher for monocytes compared to endothelial cells, whilst HA was not detected in either population (Fig. 5). These data highlight the sensitivity of the method and its suitability for GAG disaccharide analysis of low-input FACS-isolated cellular populations.

**Figure 5:**
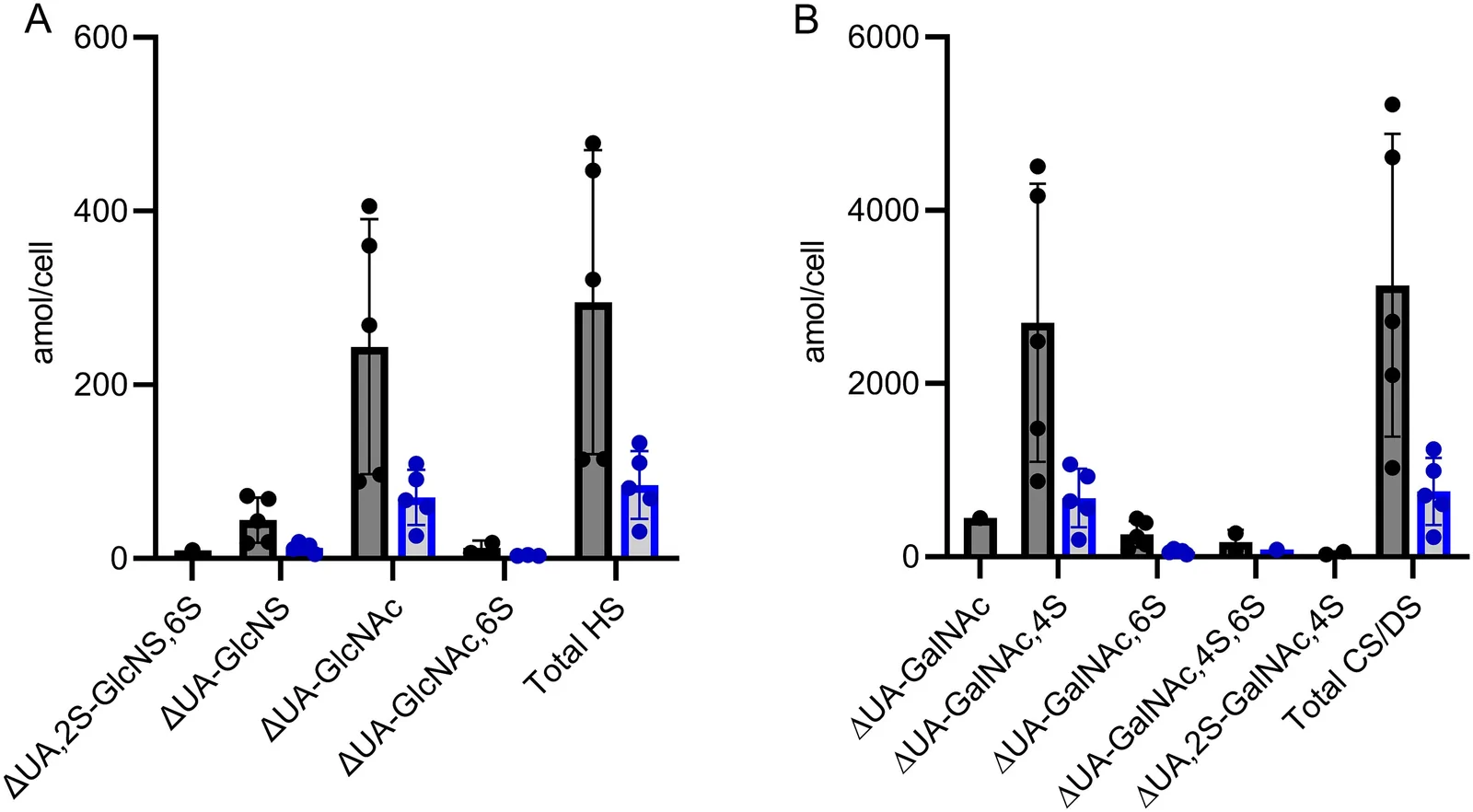
Analysis of low-input FACS-sorted cells. (A) HS and (B) CS/DS/HA disaccharide analysis of blood-derived monocytes (grey) and brain-derived endothelial cells (blue) isolated from murine tissue by FACS. *n* = 5 biological replicates.

Next, to assess application of the method to preserved biological samples, mouse kidneys were harvested from a single animal, with one frozen and one preserved by being formalin-fixed, paraffin-embedded (FFPE). Frozen tissue (~ 5 mg) was excised and FFPE scroll samples (three 25 μm sections per sample, estimated tissue volume ~ 2 mm^3^ and tissue mass ~ 2 mg, assuming a tissue density of 1 mg/mm^3^) were homogenised, and GAG disaccharides prepared and analysed. HS and CS/DS disaccharides were readily detected in both frozen and FFPE samples (Fig. 6; Supplementary Table 7). When normalised to tissue input, HS and CS/DS recoveries were slightly higher in frozen tissue than in FFPE samples, indicating a minor improvement in the efficiency of GAG recovery from frozen tissue compared to FFPE scrolls (Fig. 6A and B). HA was detected at low abundance only in frozen tissue but absent FFPE samples, which likely reflects both a lower recovery from the FFPE tissue type, and the lower amount of starting material available from the FFPE scrolls (estimated tissue mass ~ 2 mg) compared with the frozen tissue biopsies (~ 5 mg) analysed here. In both frozen and FFPE tissue, the relative abundance of GAG classes followed the order HS > CS/DS > HA, consistent with previous reports (Reine et al. 2013; Tykesson et al. 2025).

**Figure 6:**
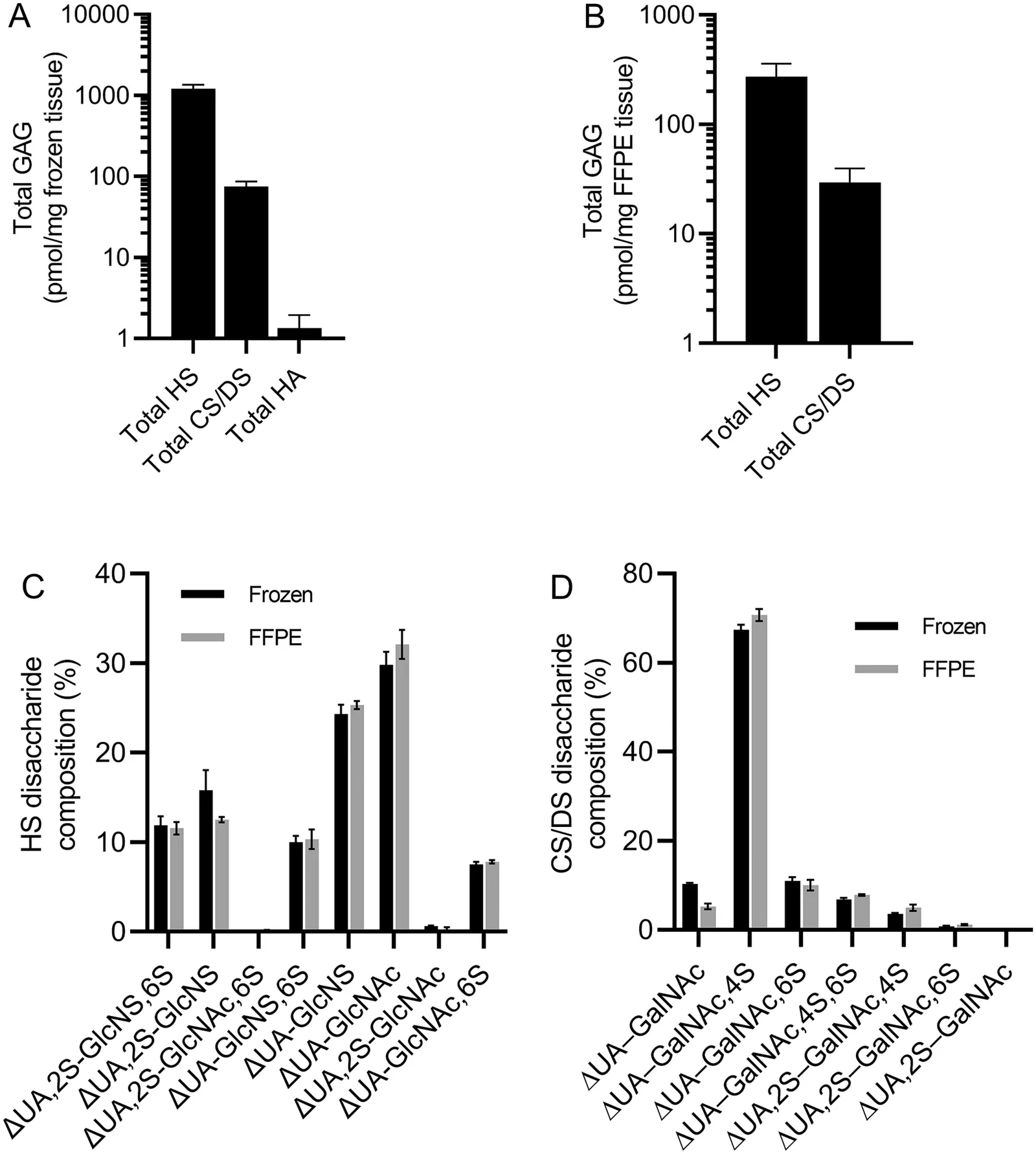
Analysis of differentially preserved biological samples. GAG disaccharide analysis was performed on frozen and FFPE mouse kidney tissue from an individual animal. Total HS, total CS/DS and total HA amounts were determined for frozen (A) and FFPE (B) tissue, and normalised by frozen tissue weights or estimated FFPE tissue weight, respectively. Disaccharide compositions of HS (C) and CS/DS (D) are shown for frozen tissue (black bars) and FFPE tissue (grey bars). *n* = 5 technical replicates.

Importantly, disaccharide composition for HS and CS/DS were highly consistent across FFPE and frozen tissue (Fig. 6C and D). The profiles observed here were in agreement with published kidney GAG compositions and lie within the range reported across previous studies, including a recent analysis of HS, CS/DS and HA (Supplementary Table 7, (Tykesson et al. 2025)). Collectively, these findings demonstrate that the workflow can successfully profile GAG disaccharides from both frozen and FFPE-preserved tissue while maintaining highly comparable compositional information across preservation formats.

### Differences in GAGs revealed across biological samples

Next, the ability of the method to reveal new biological insight was explored. We have previously demonstrated that HS levels are elevated in the serum of mice in a model of psoriasis, following shedding from the immune cell using ELISA approaches (Priestley et al. 2025). In this same study we determined that these increased HS levels in the serum were due to psoriasis driven inflammatory shedding of HS from the surface of immune cells, a process that regulates their recruitment to the inflamed skin. To further probe this interesting biology we analysed the same samples with our method and confirmed increased circulating HS in the inflamed mouse serum (Fig. 7A). Furthermore, the analysis here suggests that this change was largely due to an increase in the unsulfated HS disaccharide ∆UA-GlcNAc, providing additional information to our previous observations that may reveal further underlying mechanistic biology, e.g. changes in binding capacity of the HS shed in this inflammatory model (Ridley et al. 2023). CS/DS disaccharide analysis, which we were not able to perform in the original study, of the same samples also indicated an increase in the unsulfated CS/DS disaccharide ∆UA-GalNAc, and a slight increase in total CS/DS amounts, although the latter was not statistically significant (Fig. 7B). The amount of HA did not increase (Fig. 7B).

**Figure 7:**
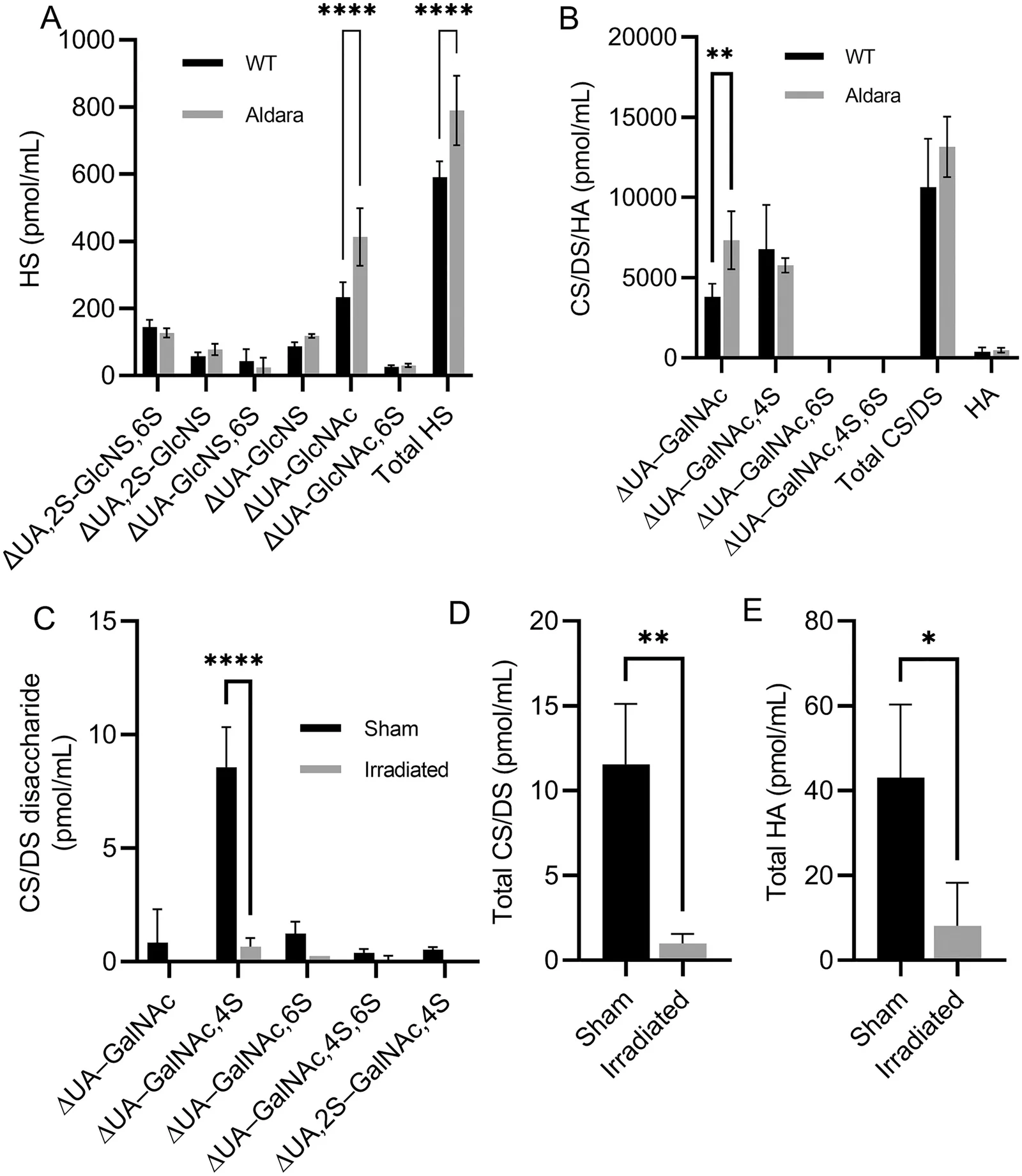
GAG changes in biological models of disease. In a mouse model of psoriasis, (A) HS and (B) CS/DS/HA disaccharides differences were detected between untreated (black) and treated (grey) mouse serum (*n* = 4 biological replicates, two-way anova). Changes to (C) CS/DS disaccharide amounts and (D) total CS/DS and HA amounts in colonic mouse mucus after sham treatment (black) or radiation (grey) (*n* = 3 biological replicates, unpaired T-test).

In another application we have previously demonstrated that a model of cancer treating intestinal irradiation disrupts the intestinal barrier and the associated immune environment (Pun et al. 2026). To extend these biological findings we applied this method to reveal that GAG biology also changes in mouse colonic mucus, which regulates the gut barrier and associated immune response (Sharpe et al. 2018), after irradiation to model normal tissue toxicity induced by abdominal radiotherapy (Fig. 7D). This revealed a drastic reduction in the amounts of HA and CS compared to sham treatment.

## Discussion

Here, we build on existing methods to develop extensively documented end-to-end biological workflows for label-free GAG disaccharide analysis that can incorporate diverse biological samples at low sample size requirements, with the goal of increasing accessibility of GAG disaccharide analysis to non-specialist users. We provide extensive step-by-step guides for each stage, enabling reproduction of the methods in their entirety. To further support method reproducibility and accessibility, we utilised sample preparation strategies with commercially available consumables, such as MWCO filters. Additionally, disaccharides were kept label-free, which served to both reduce preparation time and bypass health and safety barriers in utilising hazardous chemicals involved in some labelling strategies.

The HILIC-MS/MS employed here for detection of the label-free disaccharides provided the means to detect each GAG disaccharide via unique MS/MS fragmentation and/or chromatographic separation. Previous label-free HILIC-MS-based studies have established the analytical capability of this approach using purified GAGs (Gill et al. 2013; Poyer et al. 2021; Hirai et al. 2025), tissue sections (Turiák et al. 2018; Raghunathan et al. 2019) and more recently tissue-fragments from LMD (Mernie and Zaia 2026). Building on these foundations, the present work extends the application of label-free HILIC-MS/MS to a broader range of biological sample types (Figs. 3–6), with the aim of demonstrating its utility across diverse research and clinically relevant specimens. To further illustrate the biological relevance of the workflow beyond analytical performance alone, we also demonstrate its ability to distinguish differences between disease models (Fig. 7).

The MS/MS fragment ions identified here agree with those identified previously (Desaire and Leary 2000; Saad and Leary 2005). The overlapping HILIC retention of HS and CS/DS/HA disaccharides and lack of unique MS/MS fragments meant that the method required separate heparinase/chondroitinase ABC digestion. Whilst this is a common approach for GAG disaccharide analysis (Basu and Weiss 2023), other groups have shown that AMAC (Chang et al. 2012; Volpi et al. 2025) or 2AB-labelled GAG disaccharide approaches can be run with dual digest and a single LC–MS/MS method (Hanamatsu et al. 2023). More recently, Hirai et al. also demonstrated that HILIC-MS can be used with a single analytical method for disaccharides by combining two HILIC columns in series (Hirai et al. 2025). The workflow described here produced measurable signals for pure disaccharides at the lowest amount evaluated (0.025 pmol) for the majority of disaccharides, which is comparable to the sensitivity reported for other standard-flow HILIC-based methods, including that of Hirai et al. Although formal limits of detection were not determined, the sensitivity achieved was sufficient for the analysis of diverse biological materials, including samples available in limited quantities. Additional sensitivity has been reported using nanoflow HILIC-MS approaches (Turiák et al. 2018), although commercially available implementations of these workflows remain limited.

The approach taken here for the approximation of disaccharide amounts in biological tissues (semi-quantification) was to normalise sample peaks to a single calibration point of an equimolar disaccharide standard. Alternative methods for quantification include the utilisation of 13C-labelled disaccharide and polysaccharides (Basu et al. 2025; Wang and Liu 2025). The quantification performed here was appropriate for the relative comparison of disaccharide amounts, total GAG amounts and calculation of disaccharide composition, and was shown to be applicable to diverse sample types and a range of starting material including those in limited quantity (e.g. ~ 6,000 cells (Fig. 5), 3× FFPE scrolls and < 5 mg tissue (Fig. 6).

The robustness of the method for biological samples was demonstrated via the observation of consistency of disaccharide amounts and disaccharide composition from replicate samples of lung tissue homogenate (Fig. 3). Furthermore, the HS and CS/DS compositions observed here for mouse lung homogenates, CHO cell pellets and mouse kidney tissue were in agreement with data reported elsewhere (Figs. 3, 4 and 6; Supplementary Tables 3, 4 and 7; (Chen et al. 2018, Miller et al. 2020, Karlsson et al. 2021, Tykesson et al. 2025). The relative abundance of HS, CS/DS and HA was also broadly comparable to published findings for matched sample types. Notably, differences between bulk lung tissue and lung homogenates (Supplementary Fig. 5) suggest that tissue disruption and sample preparation strategies can influence measured GAG abundances, emphasising the importance of considering sample type when making comparisons between studies. Taken together, the reproducibility of replicate analyses and the overall agreement with published data support the accuracy of the workflow for biological samples.

In addition to confirming method robustness and accuracy, the ability of the method to analyse biological samples across a range of input amounts was explored. Using CHO cells, GAG levels correlated strongly with cell number, including down to 20,000 cells, the lowest input evaluated in this study (r^2^ > 0.99; Fig. 4). These results demonstrate the ability of the method to reliably deduce differences in both GAG disaccharide abundance and total GAG content arising from variations in sample input. Previous studies using CHO cells have reported linear quantification down to as few as 500 cells (Li et al. 2015). In the present study, emphasis was placed on demonstrating applicability across diverse biological sample types. Accordingly, the workflow was successfully applied to FACS-sorted immune and endothelial cell populations, including samples containing approximately 1,300 cells, with the most abundant disaccharides consistently detected across biological replicates (Supplementary Tables 5 and 6, Fig. 5).

In addition to FACS-sorted cells, FFPE tissue represents another important sample type for biological and clinical analyses. Strong HS and CS/DS disaccharide signals were obtained from FFPE kidney scrolls (three 25 μm sections per sample; estimated tissue volume ~ 2 mm^3^), demonstrating successful application of the workflow to preserved tissue specimens. Recent studies have further extended GAG analysis to increasingly small tissue inputs, including single FFPE sections and laser-microdissected tissue regions with volumes as low as 1.2 × 10^5^ μm^3^, enabling spatially resolved investigation of tissue substructures (Turiák et al. 2018; Raghunathan et al. 2019; Mernie and Zaia 2026). Importantly, here the disaccharide compositions obtained from FFPE kidney tissue showed good agreement with matched frozen tissue samples (Fig. 6). HS and CS/DS compositions observed were also within the range reported in recent studies (Tykesson et al. 2025; Supplementary Table 7). Collectively, these findings demonstrate that the HILIC-MS/MS workflow is applicable to preserved tissues at laboratory-relevant sample scales and generates biologically representative GAG profiles that are consistent with both frozen tissue analyses and wider literature.

The ability of the workflow to reveal biologically meaningful differences was explored using disease models. This was exemplified here by confirmation of increased HS levels in the circulation of mice from a psoriasis model and new findings of increased circulating CS levels in these mice (Priestley et al. 2025). Furthermore, in a model of radiation induced intestinal injury we also revealed changes in GAG levels of the colonic mucus that regulates intestinal barrier, and associated immune, functions (Fig. 7) (Pun et al. 2026). By studying diverse sample types, this analysis can be applied to a broad range of research contexts and provides rich disaccharide-level information to help advance the study of GAGs in complex biological systems.

Beyond the applications noted here, the workflow is also compatible with additional analyses that could further expand the structural information obtained from biological samples. Potential extensions include (a) quantification of saturated non-reducing end disaccharides to estimate GAG chain length (Gill et al. 2013; Mernie and Zaia 2026); (b) characterisation of lyase-resistant 3OS oligosaccharides to provide additional structural information on HS (Mernie et al. 2025); and (c) replacement of pronase with trypsin digestion to facilitate integration of GAG disaccharide analysis with proteomic workflows. Continued improvements in the availability of analytical standards and reference materials should further facilitate incorporation of these complementary measurements into routine workflows. Together, these complementary approaches could provide additional biological context to the compositional changes observed here. For example, estimation of GAG chain length may help distinguish whether the higher abundance of GAG disaccharides observed in blood-derived monocytes relative to tissue-derived endothelial cells reflects differences in GAG abundance, chain length, or a combination of both (Fig. 5).

We anticipate that the method and extensive guidelines reported here will combine with existing LC–MS approaches and newly developed methods, such as ToF-SIMS spatial GAG mass spectrometry, to give context to the use of existing, and improving, antibody-based detection of GAGs. Together this combination of tools will help to improve our understanding of GAG function in complex biological contexts.

## Materials and methods

### Method

Detailed step-by-step protocols of the methods in this publication are available as a collection at https://doi.org/10.17504/protocols.io.x54v956n1l3e/v1.

### Materials

GAG disaccharide standards and full-length GAGs were purchased from Iduron, Manchester, UK. Heparinases I, II and III were purchased from New England Biolabs. All other reagents were purchased from Sigma-Aldrich, except the following: calcium chloride (EMD Millipore), ammonium acetate (Avantor), magnesium chloride (Fisher Ccientific), acetonitrile (Fisher Scientific).

### Biological material and disruption

A guide for sample disruption and amount of starting material is available at https://doi.org/10.17504/protocols.io.5jyl88pwrl2w/v1.

For precision studies, mouse lung tissue was harvested after perfusion from C57BL/6 WT females aged 9–13 weeks, stored briefly at −20 °C and then processed with a Covaris CryoPREP dry pulveriser. The powdered tissue was reconstituted at 200 mg/mL in PBS and homogenised further on a Qiagen Tissue Lyser II with 5 mm stainless steel bead for 4 × 30 seconds, and supernatant used as mouse lung tissue homogenate for GAG disaccharide analysis. For comparison of bulk tissue vs homogenate, mouse lung tissue was harvested from C57BL/6 WT females aged 9–13 weeks, powdered tissue generated as above (bulk), with an aliquot taken for bead homogenisation (homogenate).

CHO-ZN GS−/− WT cells were maintained in suspension culture under constant agitation (170 rpm) at 37 °C and 5% CO_2_. The cells were in serum-free culture media using a 1:1 mix of BalanCD CHO Growth A (Irvine scientific) and EX-CELL® CD CHO Fusion (Sigma-Aldrich) media, with 2 mM GlutaMAX (GIBCO). Engineered CHO cells were cultured to a concentration of approximately 2 × 10^6^ cells/mL, before cells were centrifugated at 500× g for 3 mins, washed and centrifuged two times with PBS and stored as cell pellets at −80 °C. After thawing, cell pellets were reconstituted in 50 mM ammonium acetate pH 7.5 in a sonication bath for 20 minutes before preparation of GAG disaccharides.

Mouse kidneys were harvested from an individual C57Bl/6NTac WT male aged 19 weeks, one was snap frozen in liquid nitrogen and stored at −80 °C and the other was fixed with formalin and paraffin embedded (FFPE). The frozen kidney was processed on a Covaris CryoPREP dry pulveriser and ~ 5 mg of powered tissue used per sample replicate. FFPE tissue was sectioned into scrolls (25 μm thick curls), placed inside a 2 mL microfuge tube with a 5 mm stainless steel ball bearing and homogenised on a Qiagen Tissue Lyser II with 200 μL PBS for 4 × 30 seconds. Homogenised FFPE material was decrosslinked at 98 °C for 30 minutes. The homogenisation and decrosslinking steps were repeated once and samples spun at 300× g for 1 minute. The homogenised tissue was recovered in the PBS by pipetting and was used for GAG disaccharide analysis.

For FACS-sorted cells, mice were perfused with saline and blood was extracted through cardiac puncture with needles coated in EDTA. Blood was spun at 1200× g for 10 minutes to collect cells, which were resuspended in red blood cell lysing buffer (Sigma Aldrich) and washed in PBS. Brain hemispheres were chopped up finely in Hanks’ Balanced Salt Solution (HBSS) (Sigma-Aldrich), homogenised in a loose glass dounce homogeniser (DWK Life Sciences) and passed through a 70 μM cell strainer (Falcon). The resulting unicellular suspension was resuspended in 30% isotonic percoll (Cytiva) solution in HBSS and spun at 900× g for 25 minutes. The myelin layer and supernatant were aspirated off, and pelleted cells were retained for staining. Samples were stained with DRAQ7 viability dye (Invitrogen), incubated with Fc block (BD Biosciences) and stained with antibody cocktail (containing Ly6G, CD31, Ly6C, CD45, CD11b antibodies) prepared in flow buffer (made up of 0.5% BSA in PBS) for 30 minutes at 4 °C. Samples were washed and resuspended in flow buffer for cell sorting. Samples were sorted on a BD FACSAria™ Fusion Flow Cytometer (BD Biosciences) at 7.5 psi using a 140 μm nozzle with BD FACSDiva Software before being collected into 1.5 mL tubes. Cells were then frozen at −80 °C until used for HILIC-MS/MS. CD31+ endothelial cells were gated as DRAQ7-, CD45-, and CD31+. Ly6C+ monocytes gated as DRAQ7-, CD45+, CD11b+, Ly6G-, and Ly6C+.

The mouse psoriasis model is described in detail elsewhere (Priestley et al. 2025). Briefly, mice were anaesthetised using isoflurane and 10 mg of Aldara cream containing 5% Imiquimod (Meda Pharmaceuticals), was topically applied on both ear pinnae daily for 6 days. Blood samples were taken from mice by cardiac puncture under terminal anaesthesia and left on ice to coagulate for 4 hours. Blood was centrifuged at 15,000× g for 10 mins at 4 °C and the serum supernatants collected and frozen prior to HS analysis.

For colon irradiation studies, female C57BL/6 mice (Charles River), aged 11 weeks were irradiated as established previously (Pun et al. 2026). Mice were anaesthetised using isoflurane (2%) with oxygen (1 L/min) for up to 10 minutes throughout the irradiation period. Mice were then transferred to the Small Animal Radiation Research Platform (SARRP, Xstrahl), equipped with nose-cone isoflurane delivery, for CT scan image-guided precise X-ray irradiation. Single fraction dose (12 Gy) was applied to the abdomen of C57BL/6 or transgenic mice, using a parallel-opposed method delivering a half dose from the left side and a half from the right side of the mouse body. Care was taken to avoid the long bones and the spinal cord to protect the bone marrow, as well as other major organs near the field of irradiation. Mice were sacrificed 7 days after treatment and the outer colonic mucus layer was collected via a 2 mL PBS wash, and rocked at 4 °C for 5 hours before storage at −80 °C. Samples were thawed, centrifuged and 1 mL supernatant taken for CS/DS/HA analysis.

### Label-free GAG disaccharide generation from biological samples

Detailed step-by-step protocols for label-free GAG disaccharide generation, and vialling for HILIC-MS/MS analysis, are available at https://doi.org/10.17504/protocols.io.kqdg31yxel25/v1 and https://doi.org/10.17504/protocols.io.3byl46progo5/v1, respectively.

Briefly, biological material was digested with 1 mg/mL pronase (with 50 mM ammonium acetate and 1 μM calcium chloride) overnight at 37 °C, and heat inactivated at 98 °C for 10 minutes. DNase I was added at a final concentration of 0.4 mg/mL (with 0.5 μM magnesium chloride) and incubated at 37 °C for 1 hour. Samples were centrifuged and the supernatant filtered through Amicon 500 μL 3 kDa MWCO filters. The retentate (containing full-length GAGs liberated from proteins and DNA) was washed with ddH_2_O and concentrated to ~50 μL. For HS disaccharide generation, samples were digested with 1 μL of Heparinase I (12 U), II (4 U), III (0.7 U) enzymes (with ~ 40 mM ammonium acetate pH 7, 3 mM calcium chloride) and incubated at 30 °C overnight (units are given as per the NEB guidelines). For CS/DS/HA disaccharide generation, samples were digested with 10 mU Chondrotinase ABC and incubated overnight at 37 °C. Samples were centrifuged and spiked with 8 ng of an internal disaccharide standard (∆UA-2S GlcNCOEt-6S), in order to correct for variability in the extraction and analysis from this point. Samples were passed over an OASIS HLB SPE to remove hydrophobic contaminants. Disaccharides were lyophilised, reconstituted in 20 μL water and diluted 1 in 20 into 75% acetonitrile, 44 mM ammonium acetate for HILIC-MS/MS analysis (this provided an internal standard concentration of 20 ng/mL). For each batch of sample preparation, a positive control sample QC (e.g. lung tissue homogenate) was put through the entire protocol to ensure expected data were obtained.

### Equimolar disaccharide standard preparation

For each type of GAG (HS or CS/DS/HA) an equimolar disaccharide standard (a mixture of disaccharide standards at equal molar concentration, as defined by their UV absorption at 232 nm) was prepared as described further: https://doi.org/10.17504/protocols.io.n92ld6knxg5b/v1. The purpose of this standard was to correct for disaccharide specific variability in analysis and to provide a single point calibration for the approximation of disaccharide amount (semi-quantification). Absolute quantification would require multi-point calibration and validation within matrices, however, the semi-quantification presented here is appropriate for the relative quantification of disaccharide amounts, total GAG amounts (by summation of disaccharide amounts) and disaccharide composition (relative ratio of disaccharides compared to total GAG).

In brief, each individual disaccharide standard was reconstituted using ddH_2_O and the UV absorbance at 232 nm was determined. Correction factors were calculated by dividing the UV absorbance of each standard by the UV absorbance obtained from HD002 (HS) or CD001 (CS/DS/HA). HD002 and CD001 were selected as the reference standards due to their consistency in an initial multi-batch comparison. Corrected standard concentrations were used to generate a single equimolar stock solution consisting of 100 μM of each disaccharide standard, which was subsequently diluted to 1 μM in ddH_2_O.

The 1 μM equimolar stock solution was diluted 1 in 20 in 75% acetonitrile, 44 mM ammonium acetate and spiked with internal standard at a final concentration of 20 ng/mL (∆UA-2S GlcNCOEt-6S - to correct for variability in instrument performance)). This generated a 50 nM equimolar disaccharide standard, corresponding to 0.25 picomole of each disaccharide per 5 μL injection. The standard was used as a single-point calibrant for semi-quantitative estimation of disaccharide amounts; this injection amount corresponds to approximately 20 pmol of each disaccharide in the original extracted sample.

### HILIC-MS/MS data acquisition and data processing of GAG disaccharides

A detailed step-by-step protocol for data acquisition and processing is available at https://doi.org/10.17504/protocols.io.n92ld6keog5b/v1.

HILIC-MS/MS was carried out on a Shimadzu Nexera LC 30 AD coupled to a SCIEX 7600 ZenoTOF mass spectrometer. Separations were performed on 5 μL injections at 0.25 mL/min using an InfinityLab Poroshell 120 HILIC-Z column with dimensions of 150 × 2.1 mm, 2.7 μm particle size equipped with a guard column, and mobile phase A (25 mM ammonium acetate in water) and B (25 mM ammonium acetate in 85% acetonitrile). A column temperature of 50 °C and autosampler temperature of 5 °C were used. HS disaccharides were eluted on an LC gradient of 94–90% B in 25 min, which was achieved by running solvents at 94-94-90-5-5-94–94% B in 0-3-25-25.1-27-27.1–30 minutes. CS/DS/HA disaccharides were eluted on an LC gradient of 98–87% B in 25 minutes, which was achieved by running solvents at 98-98-87-87-5-5-98–98% B in 0-16-24-26-26.2-30-30.1–35 minutes. New columns were observed to require LC gradients to be adjusted by +/− 1–5% B.

MS data were acquired in negative mode (curtain gas, 30 psi; ion spray voltage, −4500 V; temperature, 250 °C; ESI nebuliser gas pressure, 50 psi; heater gas pressure, 70 psi). High resolution-multiple reaction monitoring of the precursors (as shown in Supplementary Tables 1 and 2) was performed to generate full scan MS/MS data.

For data processing, fragment ions to be used for quantification were selected based on their specificity at the selected retention time and their positive annotation in GlycoWorkbench (Ceroni et al. 2008) (fragment ions are listed in Supplementary Tables 1 and 2). Quantification was performed in SCIEX OS (V3.0), and summed peak area ratios (relative to the spiked internal disaccharide standard) were calculated. Each disaccharide peak area ratio was normalised to that of the equimolar disaccharide standard, to correct for disaccharide specific variability in analysis and provide a single point calibration for the approximation of disaccharide amount in the extracted sample (semi-quantification). The value is considered as semi-quantitative as (a) comparison is to a single calibration point and (b) full validation within each biological matrix has not been performed. The semi-quantification presented here is appropriate for the relative quantification of disaccharide amounts, total GAG amounts (by summation of disaccharide amounts) and disaccharide composition (relative ratio of disaccharides compared to total GAG). Semi-quantitative disaccharide amounts were only reported for peaks with a signal-to-noise ratio greater than 3 (S/*N* ≥ 3).

## Supplementary Material

Supplementary_Information_031126_cwag104

## Data Availability

Raw data for HILIC-MS analysis are available at GlycoPOST under project ID GPST000759.
