# Supplementary material for: A practical framework for label-free GAG disaccharide analysis by HILIC-MS/MS: detailed protocols for analysis of diverse biological sample types
Source: Glycobiology. 2026 Nov 6;36(12):cwag104. doi: 10.1093/glycob/cwag104 (PMC13759999; doi:10.1093/glycob/cwag104)
Supplement: Supplementary file 1 [file supplementary_information_031126_cwag104.docx]

**Supplementary Information**

**A practical framework for label-free GAG disaccharide analysis by HILIC-MS/MS: detailed protocols for analysis of diverse biological sample types**

Key words: Disaccharide analysis/ Glycosaminoglycan/HILIC-MS/MS

**Authors:** H Davies-Strickleton^1,2^*, George Taylor^2^, James Allsey^2^, Samie Dalgarno^1^,^3,^, Megan J. Priestley^4^, Isobelle Blair^1, 3^, Nabina Pun^5^, Emily Williams^1^, Magnus Nørregaard Nissen Grønset^6^, Ivy Usanksy^7^, Catherine B Lawrence^7^, Rebecca L. Miller^6^, David Knight^2^*†, Douglas P. Dyer^1,3,7^*†

**Affiliations:**

^1^Manchester Cell-Matrix Centre, Faculty of Biology, Medicine and Health, University of Manchester, United Kingdom.

^2^Biological Mass Spectrometry Core Facility, Faculty of Biology, Medicine and Health, Manchester Academic Health Science Centre, University of Manchester, Manchester, UK

^3^Lydia Becker Institute of Immunology and Inflammation, MRC-UK Centre of Research Excellence in Exposome Immunology, Faculty of Biology, Medicine and Health, University of Manchester, United Kingdom.

^4^Koch Institute for Integrative Cancer Research, Massachusetts Institute of Technology; Cambridge, MA, USA.

^5^Centre for Immuno-Oncology, Nuffield Department of Medicine, University of Oxford, Oxford, UK.

^6^Copenhagen Center for Glycocalyx Research, Department of Cellular and Molecular Medicine, Faculty of Health Sciences, University of Copenhagen, Blegdamsvej 3, DK-2200 Copenhagen N, Denmark.

^7^Division of Neuroscience, Geoffrey Jefferson Brain Research Centre Faculty of Biology, Medicine and Health, Manchester Academic Health Science Centre, The University of Manchester, Manchester, UK

* Corresponding authors. Email: [douglas.dyer@manchester.ac.uk](mailto:douglas.dyer@manchester.ac.uk), [david.knight@manchester.ac.uk](mailto:david.knight@manchester.ac.uk), [h.davies-strickleton@manchester.ac.uk](mailto:h.davies-strickleton@manchester.ac.uk)

† These authors contributed equally to this work.

Supplementary Figure 1: Mass spectra of HS disaccharide standards

Supplementary Table 1: Ion transitions (precursors and fragment ions) for HS disaccharide analysis

Supplementary Figure 2: Mass spectra of CS/DS/HA disaccharide standards

Supplementary Table 2: Ion transitions (precursors and fragment ions) for CS/DS/HA disaccharide analysis

Supplementary Figure 3: Response and linearity of HS disaccharide standards

Supplementary Figure 4: Response and linearity of CS/DS/HA disaccharide standards

Supplementary Figure 5: Comparison of GAG recovery from bulk tissue and tissue homogenate

Supplementary Table 3: Intra-sample precision of disaccharide amount, total GAG amount and disaccharide composition from lung tissue homogenate replicates (n=5)

Supplementary Table 4: Disaccharide composition of CHO cells analysed here compared to literature

Supplementary Table 5: GAG disaccharide levels obtained from the analysis of blood-derived monocytes

Supplementary Table 6: GAG disaccharide levels obtained from the analysis of brain-derived endothelial cells

Supplementary Table 7: Disaccharide composition of frozen and FFPE mouse kidney tissue (average from n=5 technical replicates)

Supplementary Figure 1: MS spectra of HS disaccharide standards. MS ion scan showing precursor ions of each disaccharide standard. Inserts show MS/MS fragment ions used.

**Supplementary Table 1: Ion transitions (precursors and fragment ions) for HS disaccharide analysis**

| **Analyte Name** | **Relative Retention Time** | **Precursor ion**  **m/z** | **Fragment ion**  **m/z** |
| --- | --- | --- | --- |
| Internal standard | 1 | 552.0336 | 472.0781 |
| ΔUA,2S-GlcNS,6S | 2.13 | 287.4784 | 96.9599 |
|  |  |  | 247.5 |
|  |  |  | 137.9865 |
|  |  |  | 357.0129 |
|  |  |  | 157.0141 |
|  |  |  | 287.4784 |
| ΔUA-GlcNS,6S | 1.61 | 247.5 | 300.0404 |
|  |  |  | 247.5 |
|  |  |  | 258.0303 |
|  |  |  | 96.96 |
|  |  |  | 157.0146 |
|  |  |  | 137.9866 |
|  |  |  | 357.0139 |
| ΔUA,2S-GlcNS | 1.46 | 247.5 | 96.96 |
|  |  |  | 247.5 |
|  |  |  | 137.9866 |
|  |  |  | 157.0143 |
|  |  |  | 357.0139 |
| ΔUA,2S-GlcNAc,6S | 1.25 | 268.5049 | 96.9602 |
|  |  |  | 157.0143 |
|  |  |  | 138.9711 |
|  |  |  | 370.046 |
|  |  |  | 268.5049 |
| ΔUA-GlcNAc,6S | 0.92 | 458.0617 | 175.0256 |
|  |  |  | 357.0148 |
|  |  |  | 300.0403 |
| ΔUA,2S-GlcNAc | 0.85 | 458.0617 | 236.9712 |
| ΔUA-GlcNS | 1.21 | 416.0514 | 137.9867 |
|  |  |  | 416.0514 |
|  |  |  | 175.0255 |
| ΔUA-GlcNAc | 0.76 | 378.1048 | 175.0251 |
|  |  |  | 157.015 |
|  |  |  | 277.0567 |

Supplementary Figure 2: MS spectra of CS/DS/HA disaccharide standards. MS ion scan showing precursor ions of each disaccharide standard. Inserts show MS/MS fragment ions.

**Supplementary Table 2: Ion transitions (precursors and fragment ions) for CS/DS/HA disaccharide analysis**

| Analyte Name | Relative Retention Time | Precursor (Q1) Mass (Da) | Fragment (Q3) Mass (Da) |
| --- | --- | --- | --- |
| Internal standard | 1 | 552.0336 | 472.0781 |
| ∆UA-(1–3)-GlcNAc | 0.72 | 378.1041 | 175.0256 |
|  |  |  | 157.0146 |
|  |  |  | 378.1045 |
| ∆UA-GalNAc | 0.85 | 378.1041 | 175.0249 |
|  |  |  | 157.0143 |
|  |  |  | 378.1045 |
| ∆UA-GalNAc,4S | 1.04 | 458.0614 | 300.0399 |
|  |  |  | 342.0504 |
|  |  |  | 175.0247 |
|  |  |  | 157.0143 |
|  |  |  | 282.029 |
|  |  |  | 458.0614 |
| ∆UA-GalNAc,6S | 0.97 | 458.0614 | 282.0288 |
|  |  |  | 175.0248 |
| ∆UA-GalNAc,4S,6S | 1.15 | 268.5053 | 282.0294 |
|  |  |  | 175.0249 |
| ∆UA,2S-GalNAc,4S | 1.13 | 268.5053 | 300.0398 |
| ∆UA,2S-GalNAc,6S | 1.04 | 268.5053 | 300.0398 |
|  |  |  | 282.0294 |
|  |  |  | 157.0146 |
|  |  |  | 96.96 |
|  |  |  | 268.5053 |
| ∆UA,2S-GalNAc | 0.95 | 458.0614 | 236.971 |

Supplementary Figure 3: Response and linearity of HS disaccharide standards**.** Pure HS disaccharide standards produced measurable signals at the lowest amount evaluated (0.025 pmol) for all analytes except ∆UA,2S-GlcNAc,6S, which was measurable at 0.25 pmol, where the analyte signal was approximately five-fold higher than a minor interference observed in the internal standard (IS) blank. No comparable interferences were observed for the other analytes.

Supplementary Figure 4: Response and linearity of CS/DS/HA disaccharide standards**.** Pure CS/DS/HA disaccharide standards produced measurable signals at the lowest amount evaluated (0.025 pmol) for all analytes.

Supplementary Figure 5: Comparison of GAG recovery from bulk tissue and tissue homogenate. Mouse lung tissue was prepared by cryoPREP into powdered tissue (bulk tissue, black bar) and by further homogenisation to recover only soluble material (homogenate, grey bars) were analysed for total GAGs recovered. N = 3 technical replicates.

**Supplementary Table 3: Intra-sample precision of disaccharide amount, total GAG amount and disaccharide composition from lung tissue homogenate replicates (n=5)**

|  |  | pmol/homogenate* | |  | Disaccharide composition %* | |  | Tykesson et al. 2025  disaccharide composition** |
| --- | --- | --- | --- | --- | --- | --- | --- | --- |
|  |  | Mean | % CV |  | Mean | % CV |  |  |
| HS disaccharide | ΔUA,2S-GlcNS,6S | 2.76 | 9 |  | 5.46 | 5 |  | 3.6 |
|  | ΔUA,2S-GlcNS | 5.90 | 2 |  | 11.71 | 8 |  | 17.2 |
|  | ΔUA,2S-GlcNAc,6S | 1.32 | 10 |  | 2.60 | 5 |  | 0.5 |
|  | ΔUA-GlcNS,6S | 1.90 | 19 |  | 3.75 | 18 |  | 2.9 |
|  | ΔUA-GlcNS | 11.77 | 6 |  | 23.30 | 1 |  | 23.8 |
|  | ΔUA-GlcNAc | 24.53 | 8 |  | 48.51 | 2 |  | 42.1 |
|  | ΔUA,2S-GlcNAc | BRT | N/A |  | BRT | N/A |  | 5.3 |
|  | ΔUA-GlcNAc,6S | 2.36 | 8 |  | 4.66 | 3 |  | 4.8 |
| CS/DS disaccharide | ∆UA-GalNAc | 11.00 | 30 |  | 26.47 | 5 |  | 9.4 |
|  | ∆UA-GalNAc,4S | 25.29 | 25 |  | 61.41 | 1 |  | 68.5 |
|  | ∆UA-GalNAc,6S | 2.99 | 26 |  | 7.25 | 6 |  | 11.7 |
|  | ∆UA-GalNAc,4S,6S | 0.75 | 19 |  | 1.83 | 14 |  | 4.2 |
|  | ∆UA,2S-GalNAc,4S | 0.75 | 22 |  | 1.85 | 18 |  | 5.3 |
|  | ∆UA,2S-GalNAc,6S | 0.48 | 17 |  | 1.18 | 15 |  | 0.9 |
|  | ∆UA,2S-GalNAc | BRT | N/A |  | BRT | N/A |  | Not reported |
|  | Total HS | 50.53 | 6 |  | N/A | N/A |  | N/A |
|  | Total CS/DS | 41.26 | 25 |  | N/A | N/A |  | N/A |
|  | Total HA | 69.51 | 24 |  | N/A | N/A |  | N/A |

BRT, below reporting threshold (S/N < 3). Semi-quantitative disaccharide amounts were only reported for peaks with S/N ≥ 3.

* Data is an average of 5 technical replicates

**Data are reported for 14 week old mice within Tykesson et al. 2025

**Supplementary Table 4: Disaccharide composition of CHO cells analysed here compared to literature**

|  |  | Davies-Strickleton et al. 2026 (1e^6^ cells)* | Chen et al. 2018** |
| --- | --- | --- | --- |
| HS disaccharide | ΔUA,2S-GlcNS,6S | 5.4 | 6.0 |
|  | ΔUA,2S-GlcNS | 9.1 | 16.0 |
|  | ΔUA,2S-GlcNAc,6S | BRT | 0.0 |
|  | ΔUA-GlcNS,6S | 1.6 | 0.9 |
|  | ΔUA-GlcNS | 24.2 | 20.0 |
|  | ΔUA-GlcNAc | 56.4 | 54.0 |
|  | ΔUA,2S-GlcNAc | 0.9 | 1.9 |
|  | ΔUA-GlcNAc,6S | 2.4 | 1.3 |
| CS/DS disaccharide | ∆UA-GalNAc | 2.0 | 3.5 |
|  | ∆UA-GalNAc,4S | 92.7 | 94.3 |
|  | ∆UA-GalNAc,6S | BRT | 0.0 |
|  | ∆UA-GalNAc,4S,6S | BRT | 0.0 |
|  | ∆UA,2S-GalNAc,4S | 5.2 | 2.2 |
|  | ∆UA,2S-GalNAc,6S | 0.1 | 0.0 |
|  | ∆UA,2S-GalNAc | BRT | 0.0 |

BRT, below reporting threshold (S/N < 3). Semi-quantitative disaccharide amounts were only reported for peaks with S/N ≥ 3.

* Data is an average of 5 technical replicates

** Data converted to % from Supplementary Table 3 within Chen et al 2018

**Supplementary Table 5: GAG disaccharide levels obtained from the analysis of blood-derived monocytes**

|  |  | Blood-derived monocytes | | | | | |
| --- | --- | --- | --- | --- | --- | --- | --- |
|  |  | *Sample 1* | *Sample 2* | *Sample 3* | *Sample 4* | *Sample 5* | Mean |
|  | *#cells analysed for HS:* | *8640* | *17027* | *7842* | *17031* | *6182* | *11344* |
| HS disaccharide amount (attomol/cell) | ΔUA,2S-GlcNS,6S | BRT | BRT | 10 | BRT | BRT | 2 |
|  | ΔUA,2S-GlcNS | BRT | BRT | BRT | BRT | BRT | BRT |
|  | ΔUA,2S-GlcNAc,6S | BRT | BRT | BRT | BRT | BRT | BRT |
|  | ΔUA-GlcNS,6S | BRT | BRT | BRT | BRT | BRT | BRT |
|  | ΔUA-GlcNS | 69 | 19 | 43 | 18 | 72 | 44 |
|  | ΔUA-GlcNAc | 360 | 89 | 269 | 97 | 406 | 244 |
|  | ΔUA,2S-GlcNAc | 0 | 0 | 0 | 0 | 0 | 0 |
|  | ΔUA-GlcNAc,6S | 18 | 7 | 0 | 0 | 0 | 5 |
|  | *#cells analysed for CS/DS/HA:* | *960* | *1892* | *871* | *1892* | *687* | *1260* |
| CS/DS disaccharide (attomol/cell) | ∆UA-GalNAc | BRT | BRT | BRT | 445 | BRT | 89 |
|  | ∆UA-GalNAc,4S | 4165 | 872 | 2486 | 1482 | 4506 | 2702 |
|  | ∆UA-GalNAc,6S | 391 | 88 | 230 | 144 | 443 | 259 |
|  | ∆UA-GalNAc,4S,6S | BRT | 67 | BRT | BRT | 272 | 169 |
|  | ∆UA,2S-GalNAc,4S | 55 | BRT | BRT | 27 | BRT | 16 |
|  | ∆UA,2S-GalNAc,6S | BRT | BRT | BRT | BRT | BRT | BRT |
|  | ∆UA,2S-GalNAc | BRT | BRT | BRT | BRT | BRT | BRT |
|  | Total HS | 447 | 115 | 321 | 114 | 478 | 295 |
|  | Total CS/DS | 4611 | 1027 | 2716 | 2097 | 5221 | 3134 |
|  | Total HA | BRT | BRT | BRT | BRT | BRT | BRT |
|  | BRT, below reporting threshold (S/N < 3). Semi-quantitative disaccharide amounts were only reported for peaks with S/N ≥ 3. For calculation of mean, data that were BRT were counted as 0. | | | | | | |

**Supplementary Table 6: GAG disaccharide levels obtained from the analysis of brain-derived endothelial cells**

|  |  | Brain-derived endothelial cells | | | | | |
| --- | --- | --- | --- | --- | --- | --- | --- |
|  |  | *Sample 1* | *Sample 2* | *Sample 3* | *Sample 4* | *Sample 5* | Mean |
|  | *#cells analysed for HS:* | *27000* | *26100* | *19800* | *14400* | *30600* | *23580* |
| HS disaccharide amount (attomol/cell) | ΔUA,2S-GlcNS,6S | BRT | BRT | BRT | BRT | BRT | BRT |
|  | ΔUA,2S-GlcNS | BRT | BRT | BRT | BRT | BRT | BRT |
|  | ΔUA,2S-GlcNAc,6S | BRT | BRT | BRT | BRT | BRT | BRT |
|  | ΔUA-GlcNS,6S | BRT | BRT | BRT | BRT | BRT | BRT |
|  | ΔUA-GlcNS | 15 | 12 | 19 | 11 | 5 | 12 |
|  | ΔUA-GlcNAc | 91 | 67 | 109 | 59 | 26 | 71 |
|  | ΔUA,2S-GlcNAc | BRT | BRT | BRT | BRT | BRT | BRT |
|  | ΔUA-GlcNAc,6S | 3 | 3 | 4 | BRT | BRT | 2 |
|  | *#cells analysed for CS/DS/HA:* | *3000* | *2900* | *2200* | *1600* | *3400* | *2620* |
| CS/DS disaccharide (attomol/cell) | ∆UA-GalNAc | BRT | BRT | BRT | BRT | BRT | BRT |
|  | ∆UA-GalNAc,4S | 1066 | 643 | 926 | 557 | 199 | 678 |
|  | ∆UA-GalNAc,6S | 94 | 66 | 67 | 51 | 26 | 61 |
|  | ∆UA-GalNAc,4S,6S | 80 | BRT | BRT | BRT | BRT | 16 |
|  | ∆UA,2S-GalNAc,4S | BRT | BRT | BRT | BRT | BRT | BRT |
|  | ∆UA,2S-GalNAc,6S | BRT | BRT | BRT | BRT | BRT | BRT |
|  | ∆UA,2S-GalNAc | BRT | BRT | BRT | BRT | BRT | BRT |
|  | Total HS | 110 | 81 | 133 | 69 | 31 | 85 |
|  | Total CS/DS | 1240 | 709 | 993 | 608 | 225 | 755 |
|  | Total HA | BRT | BRT | BRT | BRT | BRT | BRT |
|  | BRT, below reporting threshold (S/N < 3). Semi-quantitative disaccharide amounts were only reported for peaks with S/N ≥ 3. For calculation of mean, data that were BRT were counted as 0. | | | | | | |

**Supplementary Table 7: Disaccharide composition of frozen and FFPE mouse kidney tissue (average from n=5 technical replicates)**

|  |  | Mean disaccharide composition % | |  | Tykesson et al. 2025  disaccharide composition* |
| --- | --- | --- | --- | --- | --- |
|  |  | Frozen | FFPE |  |  |
| HS disaccharide | ΔUA,2S-GlcNS,6S | 11.9 | 11.6 |  | 9.3 |
|  | ΔUA,2S-GlcNS | 15.8 | 12.5 |  | 8.8 |
|  | ΔUA,2S-GlcNAc,6S | 0.1 | 0.1 |  | 0.5 |
|  | ΔUA-GlcNS,6S | 10.0 | 10.4 |  | 9.6 |
|  | ΔUA-GlcNS | 24.3 | 25.3 |  | 21.5 |
|  | ΔUA-GlcNAc | 29.8 | 32.1 |  | 37 |
|  | ΔUA,2S-GlcNAc | 0.6 | 0.2 |  | 1.3 |
|  | ΔUA-GlcNAc,6S | 7.5 | 7.8 |  | 11.9 |
| CS/DS disaccharide | ∆UA-GalNAc | 10.3 | 5.3 |  | 13.1 |
|  | ∆UA-GalNAc,4S | 67.3 | 70.7 |  | 58.2 |
|  | ∆UA-GalNAc,6S | 11.1 | 10.1 |  | 14.8 |
|  | ∆UA-GalNAc,4S,6S | 6.8 | 7.8 |  | 9.5 |
|  | ∆UA,2S-GalNAc,4S | 3.6 | 5.0 |  | 3.4 |
|  | ∆UA,2S-GalNAc,6S | 0.8 | 1.2 |  | 1.1 |
|  | ∆UA,2S-GalNAc | BRT | BRT |  | Not reported |

BRT, below reporting threshold (S/N < 3). Semi-quantitative disaccharide amounts were only reported for peaks with S/N ≥ 3.

*Data are reported for 14 week old mice within Tykesson et al. 2025
